# Renal Memo1 Differentially Regulates the Expression of Vitamin D-Dependent Distal Renal Tubular Calcium Transporters

**DOI:** 10.3389/fphys.2018.00874

**Published:** 2018-07-09

**Authors:** Matthias B. Moor, Barbara Haenzi, Finola Legrand, Robert Koesters, Nancy E. Hynes, Olivier Bonny

**Affiliations:** ^1^Department of Pharmacology and Toxicology, University of Lausanne, Lausanne, Switzerland; ^2^Department of Nephrology, Hôpital Tenon, Université Pierre et Marie Curie, Paris, France; ^3^Friedrich Miescher Institute for Biomedical Research, Basel, Switzerland; ^4^Service of Nephrology, Department of Medicine, Lausanne University Hospital, Lausanne, Switzerland

**Keywords:** Memo, calcium transport, NCX1, TRPV5, vitamin D3, FGF23

## Abstract

Ablation of the Mediator of ErbB2-driven Cell Motility 1 (Memo1) in mice altered calcium homeostasis and renal calcium transporter abundance by an unknown mechanism. Here, we investigated the role of intrarenal Memo in renal calcium handling. We have generated a mouse model of inducible kidney-specific *Memo1* deletion. The Memo-deficient mice showed normal serum concentration and urinary excretion of calcium and phosphate, but elevated serum FGF23 concentration. They displayed elevated gene expression and protein abundance of the distal renal calcium transporters NCX1, TRPV5, and calbindin D28k. In addition, Claudin 14 gene expression was increased. When the mice were challenged by a vitamin D deficient diet, serum FGF23 concentration and TRPV5 membrane abundance were decreased, but NCX1 abundance remained increased. Collectively, renal distal calcium transport proteins (TRPV5 and Calbindin-D28k) in this model were altered by Memo- and vitamin-D dependent mechanisms, except for NCX1 which was vitamin D-independent. These findings highlight the existence of distinct regulatory mechanisms affecting TRPV5 and NCX1 membrane expression *in vivo*.

## Introduction

The Mediator of ErbB2-driven Cell Motility (Memo) was discovered in a screen for proteins binding to the phosphorylated ErbB2 receptor (Marone et al., 2004). The *Memo1* gene is highly conserved and ubiquitously expressed in cytoplasma and nucleus of mammalian tissues (Schlatter et al., 2012; Haenzi et al., 2014). Memo is a redox enzyme with unknown physiological substrate and is structurally resembling bacterial non-heme iron deoxygenases (Qiu et al., 2008; MacDonald et al., 2014).

Constitutive *Memo1* deletion caused embryonic lethality in mice (Kondo et al., 2014). We have previously reported a phenotype with traits resembling human aging and altered mineral homeostasis in mice subjected to conditional postnatal deletion of *Memo1* exon 2 (Memo cKO) in the whole body (Haenzi et al., 2014). In particular, these mice displayed increased calcemia and 1,25(OH)_2_-vitamin D_3_ levels, suppressed parathyroid hormone (PTH) levels, and had a trend to higher serum concentrations of fibroblast growth factor FGF23. Gene expression of calcium transport proteins was increased in the distal convoluted/connecting tubule (DCT-CNT), the nephron segment responsible for hormone-sensitive transcellular calcium reabsorption (Haenzi et al., 2014). Phosphate transporters showed discrete alterations in the kidney of Memo cKO mice, with altered transcription of *SLC34a3* and a different cleavage of NaPi2a protein. However, phosphatemia was unchanged between Memo null mice and controls (Haenzi et al., 2014). Memo cKO mice display also a specific bone phenotype, resembling partially hypophosphatasia (Moor et al., 2018) and pointing to a role of Memo in the assembly of active alkaline phosphatase dimers.

In addition to these findings, Memo cKO mice showed hypersensitivity to insulin, and signs of premature aging: *Memo1* deletion caused alopecia, hair graying, kyphosis, infertility, atrophy of subcutaneous fat, and Memo cKO mice have a shortened life span.

Eventually, although direct *in vivo* evidence is yet to be established, results from cellular studies have shown that Memo modulates cellular signaling responses induced by several growth factors including FGF2 and FGF23 (Marone et al., 2004; Haenzi et al., 2014; Frei et al., 2016). Accordingly, the Memo null mouse display a phenotype that resembles the one found in mice deleted for *FGF23* (Shimada et al., 2004) or for *Klotho* (Kuro-o et al., 1997), suggesting that the three genes may act in a common signaling pathway. Interestingly, the partially overlapping *Klotho* and *Fgf23* loss-of-function mouse phenotypes are largely dependent on vitamin D (Tsujikawa et al., 2003; Razzaque et al., 2006; Hesse et al., 2007; Stubbs et al., 2007; Ohnishi et al., 2009; Anour et al., 2012; Andrukhova et al., 2014).

The kidney is the principal organ expressing Klotho (Kuro-o et al., 1997) and exerting Klotho's purported main function (Lindberg et al., 2014), i.e., acting as a co-receptor for FGF23 (Urakawa et al., 2006). Both Klotho (Alexander et al., 2009) and FGF23 (Andrukhova et al., 2014) assist in precisely regulating not only phosphaturia but also renal calcium handling.

In the kidney, calcium reabsorption depends on the activity of several tubular segments, with paracellular reabsorption in the proximal tubule and in the thick ascending limb and final transcellular reabsorption in the distal convoluted and connecting tubules (Moor and Bonny, 2016). In the latter, calcium enters the cell through apical channels TRPV5, binds intracellularly to calbindin D28k and exits the cell at the basolateral side through either the calcium ATP-ase PMCA4 or the sodium-calcium exchanger NCX1. Vitamin D is a potent regulator of distal tubular calcium reabsorption.

For all these reasons, we hypothesized that Memo affects mineral homeostasis via an intrarenal process and in a manner possibly influenced by vitamin D. In the present study, we have established an inducible kidney-specific Memo KO (Memo kKO) mouse model, and we describe altered renal mineral transporters expression in these mice, partially dependent on vitamin D presence.

## Methods

### Animal studies

All animal experimental protocols were approved by the veterinary service of the Canton de Vaud, Switzerland.

To obtain an inducible Memo KO mouse model, mice floxed for exon 2 of the *Memo1* gene (Haenzi et al., 2014) backcrossed in the C57BL/6J background over at least 10 generations were crossed with [B6.Cg-Tg(Pax8 rtTA2S^*^M2)1Koes/J] mice (Traykova-Brauch et al., 2008) and with LC-1 Cre mice carrying the Cre recombinase under a Ptet bi-1 promoter (Schönig et al., 2002) to obtain an inducible kidney-specific loss of Memo1 mouse model. Genotypes were determined by PCR of ear punch biopsy or tail DNA using the following primers: *Memo1* forward 5′-*CCCTCTCATCTGGCTTGGTA*-3′, *Memo1* reverse 5′- *GCTGCATATGCTCACAAAGG*-3′, Pax8 rtTA forward 5′-*CCATGTCTAGACTGGACAAGA*-3′, Pax8 rtTA reverse 5′-*CTCCAGGCCACATATGATTAG*-3′, Cre forward 5′-*AGGTTCGTGCACTCATGGA*-3′, Cre reverse 5′-*TCACCAGTTTAGTTACCC*-3′.

Male *Memo1* floxed mice carrying the Pax8 rtTA transgene and the LC-1 Cre were treated with 0.2 mg/mL doxycycline hyclate (Sigma) in 2% sucrose in drinking water starting at age 25 to 30 days over 14 days to induce kidney-specific loss of Memo. Male littermates negative for either Pax8, LC1-Cre, or both transgenes underwent the same doxycycline treatment and served as controls, after their phenotype has been checked.

Mice were fed a standard laboratory chow (Nafnag TS3242, Kliba) containing calcium 1%, phosphorus 0.65%, magnesium 0.23%, vitamin D 1,600 IU/kg unless specifically stated otherwise. For vitamin D depletion studies, mice were fed experimental diets containing either 0 IU/kg (vitamin D-deficient diet, VDD) (Altromin GmbH C1017; calcium 0.95%, phosphorus 0.8%, magnesium 0.07%,) or a nearly identical control diet containing 500 IU/kg vitamin D (Altromin GmbH C1000; calcium 0.93%, phosphorus 0.8%, magnesium 0.07%). Mice of both genotypes were fed these diets cage-wise starting at weaning age after randomization by flipping a coin. Weight of mice fed these diets was monitored twice weekly. After 5 weeks on experimental diets, mice were put in metabolic cages 3600M021 (Tecniplast) under 12:12 h light/dark conditions. Animals were accustomed to cages for 2 days prior to measurements and had free access to food and water. Mice were anesthetized with ketamine/xylazine and bled by orbital puncture followed by cervical dislocation.

### Metabolic and endocrine studies

Electrolytes were measured by the central laboratory of the Lausanne University Hospital: Total calcium using the NM-BAPTA method, phosphate by the phosphomolybdate method, magnesium by the xylidyl blue method, albumin by the bromocresol green method, and creatinine by the modified Jaffé method. Sodium and potassium were measured using a flame photometer 943 (Instrumentation Laboratory), osmolality by an osmometer 2020 (Advanced Instrumentation, Inc.), pH using a pH-meter (Metrohm 6.0224.100). ELISA kits were used according to manufacturer's instructions: FGF23 (Kainos Japan CY-4000), PTH (Immutopics 60-2305), 1,25(OH)_2_-vitamin D EIA (ImmunoDiagnostic Systems AC-62F1). Alkaline phosphatase activity was measured using a colorimetric kit (Abcam ab83369).

### Gene expression

RNA was extracted using TRI reagent (Applied Biosystems by Life Technologies), quantified using Nanodrop (Nanodrop 2000, Thermo Fisher Scientific, Waltham, MA, USA) and reverse transcribed using PrimeScript RT (Takara Bio Inc, Otsu, Japan). qPCR was performed with SYBR Green (Applied Biosystems by Life Technologies) on a 7500 Fast machine (Applied Biosystems). Samples were run in triplicates, and *Actb* or *Gapdh* were used as house-keeping genes. The delta-delta CT method was applied for relative quantification. Melting curves were obtained for each run. Primers were ordered from Microsynth (Switzerland), and sequences are shown in Table 1. All amplified products were run on agarose gels to verify proper amplification.

**Table 1:** Primers used for qPCR.

**Oligonucleotide**	**5^′^-sequence-3^′^**
Memo exon2 forward	AGG ACC TCA GCT GAA CGC T
Memo exon2 reverse	GGC TCT AGC AGG TCT TTT CG
Slc8a1 forward	AGAGCTCGAATTCCAGAACGATG
Slc8a1 reverse	TTGGTTCCTCAAGCACAAGGGAG
Trpv5 forward	TCCTTTGTCCAGGACTACATCCCT
Trpv5 reverse	TCAAATGTCCCAGGGTGTTTCG
Calb1 forward	AACTGACAGAGATGGCCAGGTTA
Calb1 reverse	TGAACTCTTTCCCACACATTTTGAT
Atp2b4 forward	CTTAATGGACCTGCGAAAGC
Atp2b4 reverse	ATCTGCAGGGTTCCCAGATA
beta-actin forward	GTC CAC CTT CCA GCA GAT GT
beta-actin reverse	AGT CCG CCT AGA AGC ACT TGC
Gapdh forward	CCA CCC AGA AGA CTG TGG AT
Gapdh reverse	CAC ATT GGG GGT AGG AAC AC
Slc34a1 forward	TCACAGTCTCATTCGGATTTGG
Slc34a1 reverse	GGCCTCTACCCTGGACATAGAA
Slc34a3 forward	CCTACCCCCTCTTCTTGGGT
Slc34a3 reverse	AGAGCAACCTGAACTGCGAA
Klotho forward	TGTATGTGACAGCCAATG
Klotho reverse	GAATACGCAAAGTAGCCACAAAGG
Cyp27b1 forward	ATGTTTGCCTTTGCCCAGA
Cyp27b1 reverse	GACGGCATATCCTCCTCAGG
Cyp24a1 forward	GAAGATGTGAGGAATATGCCCTATTT
Cyp24a1 reverse	CCGAGTTGTGAATGGCACACT
Cldn2 forward	AAG GTG CTG CTGAGG GTA GA
Cldn2 reverse	AGT GGC AGA GATGGG ATT TG
Slc9a3 forward	CTT CAA ATG GCA CCA CGT CC
Slc9a3 reverse	AAT AGG GGG CAGCAG GTA GA
Cldn14 forward	ACC CTG CTC TGC TTA TCC
Cldn14 reverse	GCA CGG TTG TCC TTG TAG
Cldn16 forward	CAAACGCTTTTGATGGGATTC
Cldn16 reverse	TTTGTGGGTCATCAGGTAGG
Cldn19 forward	CGGGCAGGTGCAATGCAAAC
Cldn19 reverse	CAGGAGACAGCAGTCAAAGTA
Slc12a1 forward	TGG TTC AGC TGACAG GGT TG
Slc12a1 reverse	ATT GGC CTG AGCGTA GTT GT

### Protein analyses

For immunoblotting, proteins from tissue whole lysates were extracted in NP-40 buffer (50 mM HEPES pH 7.4; 150 mM NaCl; 25 mM NaF; 5 mM EGTA; 1 mM EDTA; 1% Nonidet P-40; 2M Na ortho-vanadate and 1 mM DTT supplied with 10 μg/L leupeptin (Applichem by Axonlab), 10 μg/L aprotinin, 1 mM PMSF) and lyzed by metal beads. Homogenates were spun down.

For membrane protein enrichment, kidneys were lyzed in sucrose buffer (250 mM sucrose, 150 mM NaCl, 30 mM Tris pH 7.5, 10 μg/L leupeptin, 10 μg/L aprotinin, 1 mM PMSF and Pepstatin) and homogenized using a Polytron (Kinematica AG), then spun down 2 × 10 min at 1,000 g. Supernatants were spun down at 100,000 g for 1 h; pellets were resuspended in 75 μL sucrose buffer. 20–50 μg of proteins were denaturated in Laemmli buffer containing a final concentration of 2% beta-mercaptoethanol.

Brush border membrane vesicle-enriched protein was prepared in a procedure adapted from Biber et al. (2007). In brief, 400 μL mannitol-D buffer (300 mM mannitol-D, 5 mM EGTA, 12 mM Tris, pH adjusted to 7.1) containing protease inhibitors (Complete Mini tablet, Roche, Kaiseraugst, Switzerland) was added to kidneys halves prior to homogenization with metal beads in a tissue lyser (Qiagen). 0.56 mL of ice cold H_2_O and 12 μL of MgCl_2_ 1M (final concentration 12 mM) were added, followed by 15 min incubation. Lysates were spun down 15 min at 1,500 g, and supernatants were spun down 30 min at 25,000 g. Pellets were resuspended in 200 μL mannitol-D buffer.

Proteins were separated by 7, 10, or 13% SDS-PAGE, transferred on nitrocellulose (PROTRAN, Whatman) or PVDF (BioRad) membranes, and stained with Ponceau S. Membranes were blocked in nonfat dried milk 5%-TBST and incubated with primary antibodies against Memo [1:2000, produced in-house (Haenzi et al., 2014)], NCX1 [1:1000, produced in-house (Thurneysen et al., 2002)], TRPV5 [1:50, produced in-house (van der Hagen et al., 2014)], NaPi2a [1:4000 (Custer et al., 1994)] and NaPi2c [1:3000 (Nowik et al., 2008), both anti-NaPi2a and -NaPi2c provided by Prof. Carsten Wagner, Institute of Physiology, University of Zurich], all PMCA isoforms (anti-pan PMCA) (1:500, Sigma A7952), NCC (1:500, Millipore AB3553), calbindin D28K (1:1000, Sigma C7354), actin (1:2000, Sigma A2066) followed by anti-mouse or anti-rabbit horseradish peroxidase-conjugated secondary antibodies (1:10000, Milian Analytica 115-035-003 and 111-035-003) and exposure using Fusion Solo (Witec). Densitometric quantification of protein bands was obtained using ImageJ (1.48 v) with actin as loading control for whole-tissue lysates and Ponceau S for membrane or brush border membrane vesicles-enriched protein fractions. Contrast and brightness of entire images were adjusted after quantification of bands.

### Histology

Paraffin-embedded sections of paraformaldehyde-fixed kidney sections underwent standard staining with hematoxylin and eosin. Images were acquired using identical acquisition settings.

### Immunofluorescence

Immunofluorescence was performed on tissue cryosections by washing with PBS-Triton 0.2% for 15 min, blocking 1 h in PBS-Triton 0.2%-goat serum 10% and incubating with primary antibodies recognizing Memo (1:100, Sigma HPA 042603), TRPV5 (1:500, produced in-house, van der Hagen et al., 2014) or NCX1 (1:400, produced in-house, Thurneysen et al., 2002). Subsequently, sections were incubated with fluorophore-coated anti-rabbit Alexa fluor 488 (A11008) and anti-mouse Alexa fluor 555 (A28180) antibodies (all 1:1000, purchased at Sigma) and DAPI. Images were acquired on an LSM 780 Confocal Microscope (Zeiss), using identical acquisition settings for both genotypes.

### Micro-computed tomography

Vertebral blocks kept in ethanol 70% were scanned by a 1076 Skyscan machine at voxel size 18 μm, filter AI 0.5 mm, exposure of 1,180 ms, voltage 63 kV and 166 μA of current. A BMD standard curve was obtained using 0.25 and 0.75 g/cm^3^ calcium-phosphate standards with 2 mm diameter (Skyscan). Images were reconstructed using NRecon Version 1.6.9.3 (SkyScan) and morphometry obtained by CTAn Version 1.13.2.1 (SkyScan 2003-11, Bruker 2012-13). L5 vertebral body trabecular regions were interpolated between 3 manually selected elliptic planes. Morphometry was obtained within grayscale thresholds 80/255 using 3D techniques.

### Data analysis

Data from experiments with 2 independent groups were analyzed by unpaired *t*-test. A repeated measures ANOVA was used to compare body weight data from 4 experimental groups with repeated measurements. For analysis of 2 sources of variability in 4 experimental groups and their interaction (effects of genotype, diet, and interaction between genotype and diet), two-way ANOVA were calculated using GraphPad PRISM 5.03. Two-sided *p*-values < 0.05 were considered significant.

## Results

### Generation of inducible renal tubule-specific deletion of *Memo1*

**Figure 1:**
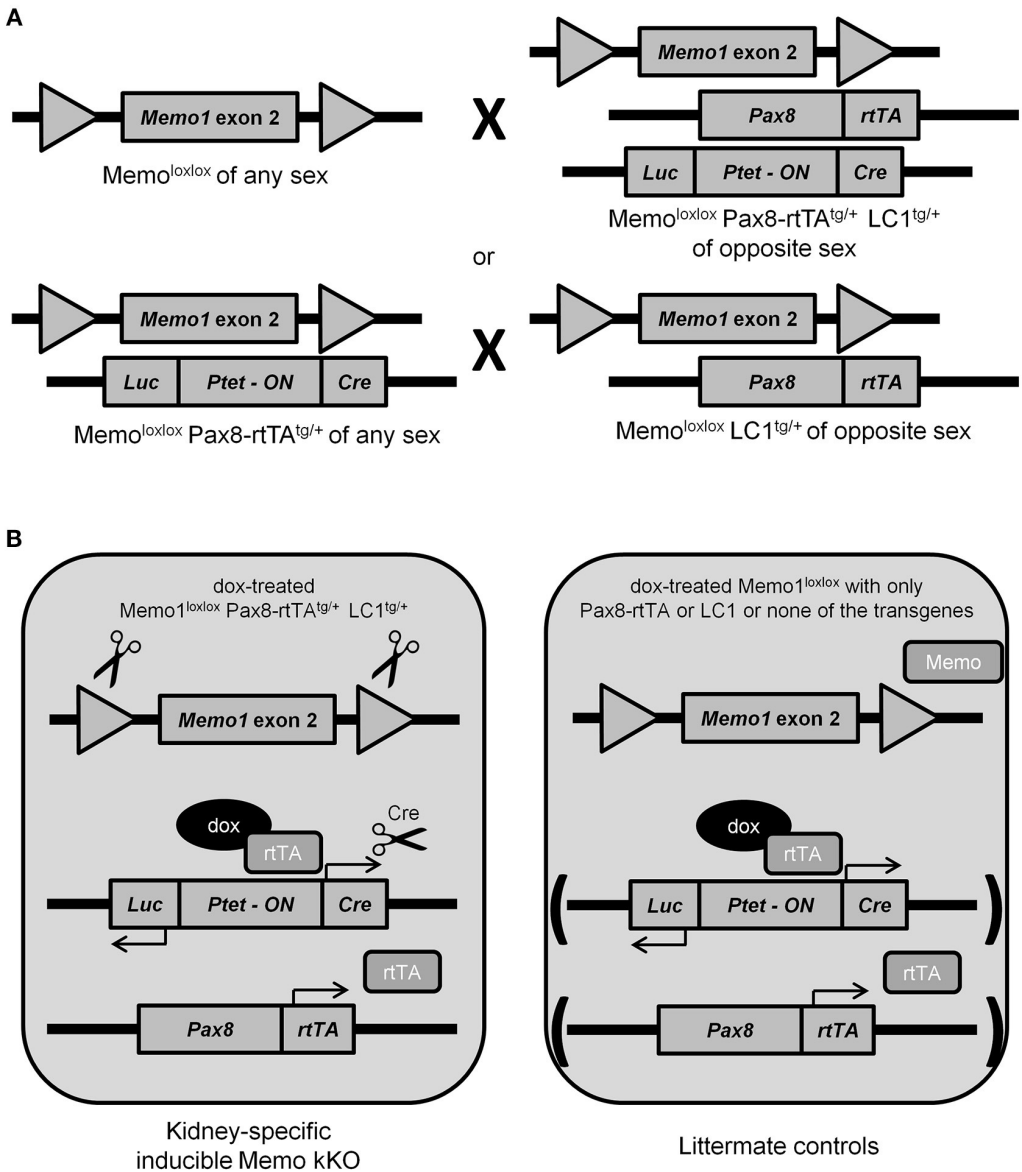
Generation of renal-specific inducible Memo kKO mice. Using two breeding strategies as depicted, Memo kKO mice were generated using both *Memo1* floxed-exon2-alleles-carrying mice and mice carrying Pax8-rtTA^tg/+^ and LC1^tg/+^ transgenes **(A)**. Male Memo kKO mice (left) and littermate controls lacking one or both of Pax8-rtTA and LC1 transgenes (right) were treated with low-dose doxycycline over 2 weeks starting at age 25–30 days **(B)**.

Previous results from our laboratory and from collaborating groups have shown disturbed mineral homeostasis and related renal transporter expression in mice after induced-Memo ablation in the whole body, using the ubiquitous actin promoter driving the cre-recombinase activity in Memo1 floxed mice (Haenzi et al., 2014). To describe the role of Memo in mineral homeostasis at the single-organ level, we now established an inducible renal-specific model of *Memo1* deletion. This was obtained by breeding Pax8-rtTA and LC1 transgenes to mice with floxed *Memo1* exon 2 (Figure 1A) and treating the resulting animals with a transient low-dose regimen of doxycycline hyclate 0.2 mg/mL in drinking water after weaning (Figure 1B). Mouse pups were born at the expected genotype ratio, indicating that the triple construct did not affect embryonic development. Upon treatment with doxycycline, body weight (Supplemental Figure) and 24 h-diuresis (Table 2) of mice of all genotypes was comparable, and no signs of premature aging or altered general appearance were observed.

**Table 2:** Serum and urine analyses of Memo kKO mice.

**Genotype**	**Control**	**kKO**	***t*-test**
	**Mean ± SD**	**Mean ± SD**	***p-*value**
**SERUM ANALYSES**			
n =	4 × 4 pooled	4 × 4 pooled	
Calcium (mM)	2.32 ± 0.02	2.34 ± 0.03	0.216
Albumin (g/L)	33.5 ± 1.91	34.25 ± 3.2	0.702
Corrected calcium (mM)	2.26 ± 0.04	2.27 ± 0.04	0.748
Phosphate (mM)	2.36 ± 0.13	2.51 ± 0.19	0.225
Sodium (mM)	154.75 ± 0.84	155.08 ± 1.54	0.724
Potassium (mM)	5.68 ± 0.33	5.46 ± 0.31	0.366
Creatinine (μM)	13.78 ± 3.67	14.70 ± 2.6	0.695
**SPOT URINE ANALYSIS**			
n =	4 × 4 pooled	4 × 4 pooled	
Calcium/creatinine (mM/ μM)	0.53 ± 0.48	0.52 ± 0.15	0.974
Phosphate/creatinine (mM/ μM)	13.50 ± 2.90	13.12 ± 3.50	0.875
Sodium/creatinine (mM/ μM)	35.73 ± 15.71	20.16 ± 10.6	0.152
Potassium/creatinine (mM/ μM)	65.75 ± 17.67	50.32 ± 12.2	0.201
FE-calcium (%)	0.70 ± 0.70	0.68 ± 0.27	0.967
FE-phosphate (%)	8.16 ± 3.83	7.71 ± 2.59	0.850
FE-sodium (%)	0.34 ± 0.20	0.20 ± 0.12	0.271
FE-potassium (%)	16.3 ± 7.59	13.56 ± 4.19	0.552
Osmolality (mOsm/kg)	2182.5 ± 483.099	1960.75 ± 306.615	0.468
pH	5.73 ± 0.14329	5.65 ± 0.14095	0.456
**24 H URINE ANALYSIS**			
n =	8	6	
Urine excretion rate (mg/g BW/24 h)	0.043 ± 0.025	0.067 ± 0.019	0.081

*SD, standard deviation; FE, fractional excretion; BW, body weight. Serum and spot urines were pooled by 4 (results from 16 male animals)*.

**Figure 2:**
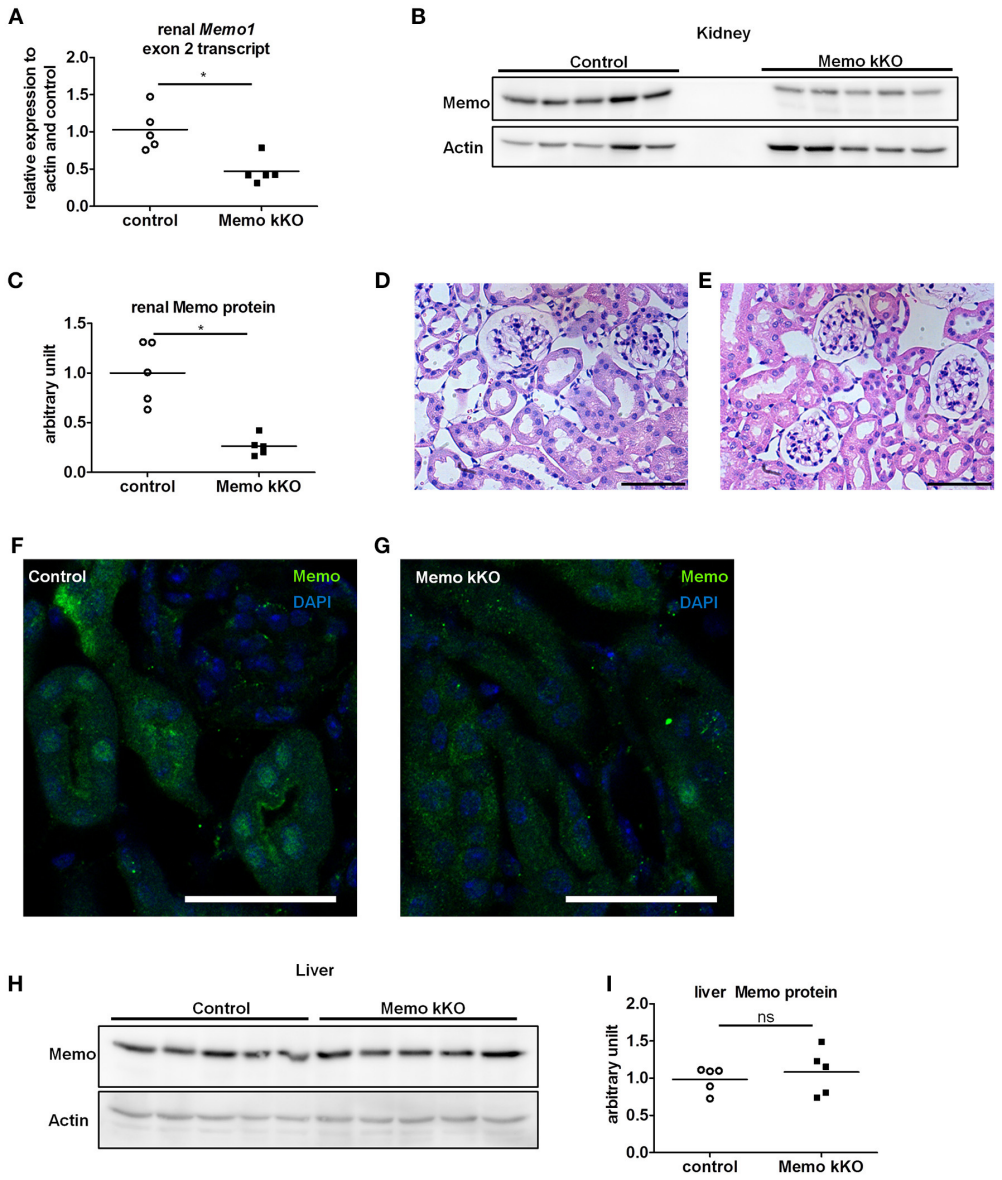
Efficient and kidney-specific Memo ablation in Memo kKO. qPCR **(A)** and Western blotting **(B)** revealed reduced renal *Memo1* exon2 transcripts and Memo protein abundance in Memo kKO; protein quantification is shown in **(C)**. Paraffin-embedded kidney sections stained with hematoxylin and eosin of control **(D)** and Memo kKO **(E)** animals show comparable morphology; scale bars are 50 μm. Immunofluorescence of renal tubuli revealed a nuclear Memo protein expression in controls **(F)**, which was diminished in Memo kKO **(G)** (green: Memo, blue: DAPI, scale bar: 40 μm). Liver Memo protein quantities were comparable between both genotypes **(H)**, quantification in **(I)**. *p < 0.05 (*t*-test); ns, not significant. *n* = 5 **(A–C, H–I)** and *n* = 3 **(D–G)** per genotype.

We observed a decrease in *Memo1* exon 2 transcripts by 54% in RNA from total kidney of Memo kKO mice (Figure 2A). Renal Memo protein levels were reduced to 26% compared to kidney from control animals (Figure 2B, quantification in Figure 2C).

To identify residual Memo protein localization, we next performed renal histology analysis. First, standard hematoxylin and eosin staining was performed and found to be comparable between controls and Memo kKO animals (Figures 2D,E) and showed normal renal histological appearance.

Next, we performed immunofluorescence and found a tubular nuclear expression pattern for Memo protein in controls (Figure 2F) similar to what was previously described (Haenzi et al., 2014). Of note, no glomerular expression was visible. In Memo kKO, tubular Memo staining was strongly decreased (Figure 2G). Finally, we verified the organ specificity of the recombination by Western blot performed with liver extracts. We could not detect a difference in Memo protein abundance between the two genotypes (Figure 2H, quantification in Figure 2I), indicating a sufficient renal specificity of the chosen recombination approach.

### Altered renal calcium handling in inducible renal tubule-specific deletion of *Memo1*

Serum and spot urines of Memo kKO and controls were collected and results of the analyses are shown in Table 2. They revealed no apparent differences in serum calcium concentration or urinary calcium excretion between the genotypes. However, final urine calcium concentration depends on the activity of several tubular segments that may cross-react and compensate for each other. The distal convoluted and connecting tubules (DCT-CNT) is the last segment in which definitive calcium excretion is regulated and we assessed expression of renal calcium transporters in these segments.

**Figure 3:**
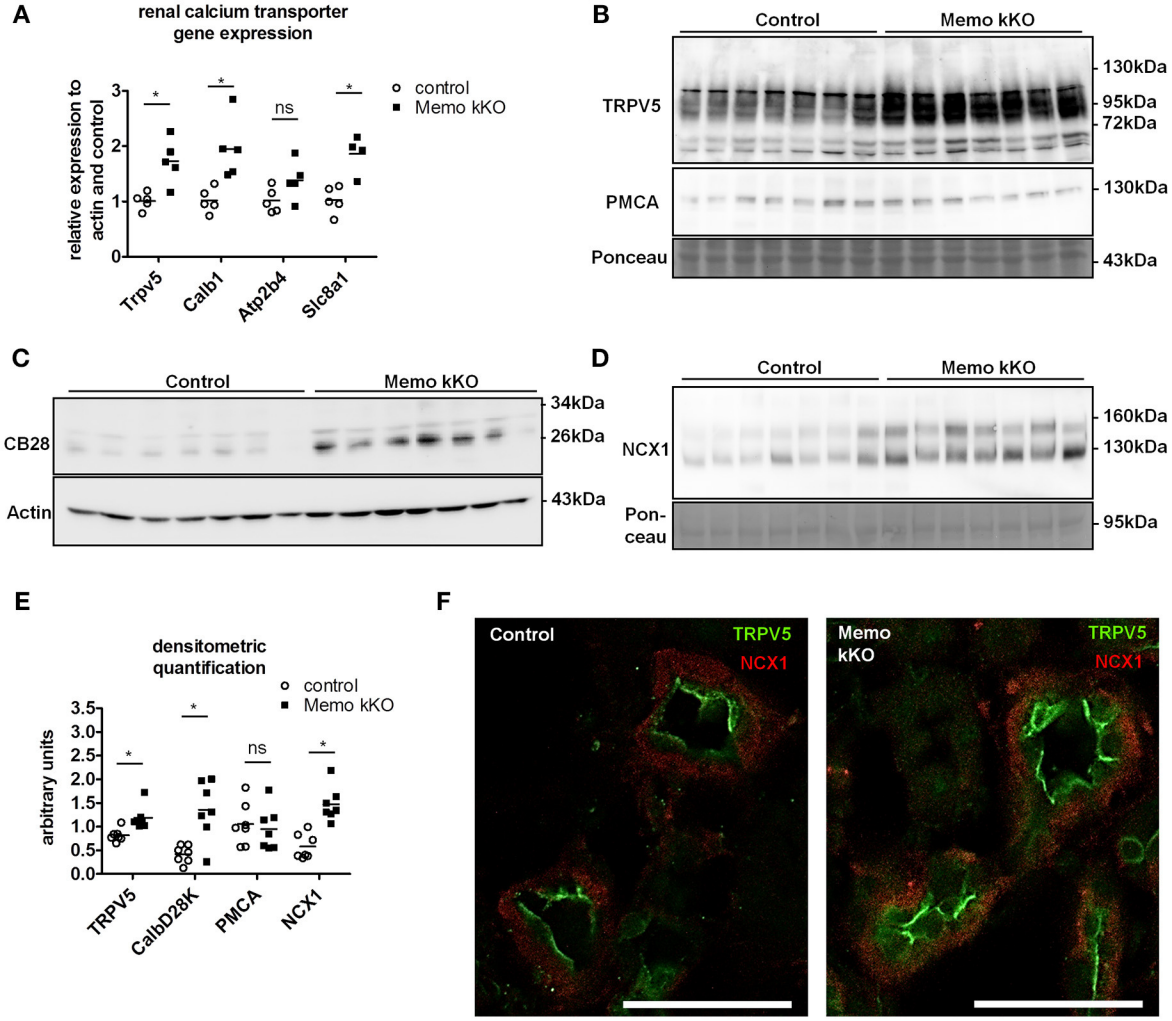
Increased renal calcium transport proteins in inducible kidney-specific Memo KO mice (kKO). Gene expression of renal calcium transporters was assessed by qPCR **(A)**. Renal membrane protein preparations **(B, D)** and whole kidney lysates **(C)** were used for Western blotting of proteins involved in calcium transport (TRPV5, PMCA, CB28 and NCX1). CB28, calbindin D28K. Densitometric quantification of **(B–D)** is shown in **(E)**. Immunofluorescence analysis of renal tubular NCX1 (red) and TRPV5 (green) protein in control and Memo kKO mice is shown **(F)**. Scale bars, 40 μm. ^*^, *p* < 0.05 (*t*-test); ns, not significant. *n* = 3 per genotype **(F)**.

Gene expression of *Trpv5* coding for the apical Transient Receptor Potential cation channel, subfamily V, member 5 and *Calb1* coding for intracellular calbindin D28K were increased in Memo kKO (Figure 3A) compared to controls. Similarly, transcripts *Slc8a1* coding for the basolateral sodium calcium exchanger (NCX)1 were increased in Memo kKO (Figure 3A). *Atp2b4* coding for the basolateral plasma membrane Ca^2+^-ATPase (PMCA) 4 was unaffected by *Memo1* deletion (Figure 3A).

In accordance with mRNA expression levels, membrane abundance of TRPV5 and NCX1 proteins and cytoplasmic calbindin D28K protein abundance were increased in Memo kKO compared to controls (Figures 3B–D). PMCA was unchanged (Figure 3B). All quantifications are shown in Figure 3E.

In addition to Western blot, distributions of renal TRPV5 channel and NCX1 protein were assessed by immunofluorescence, showing an increased abundance of both proteins in kidney sections of Memo kKO (Figure 3F).

### Discrete alterations in other nephron segments in memo kKo, and no evidence of DCT-CNT hyperplasia

**Figure 4:**
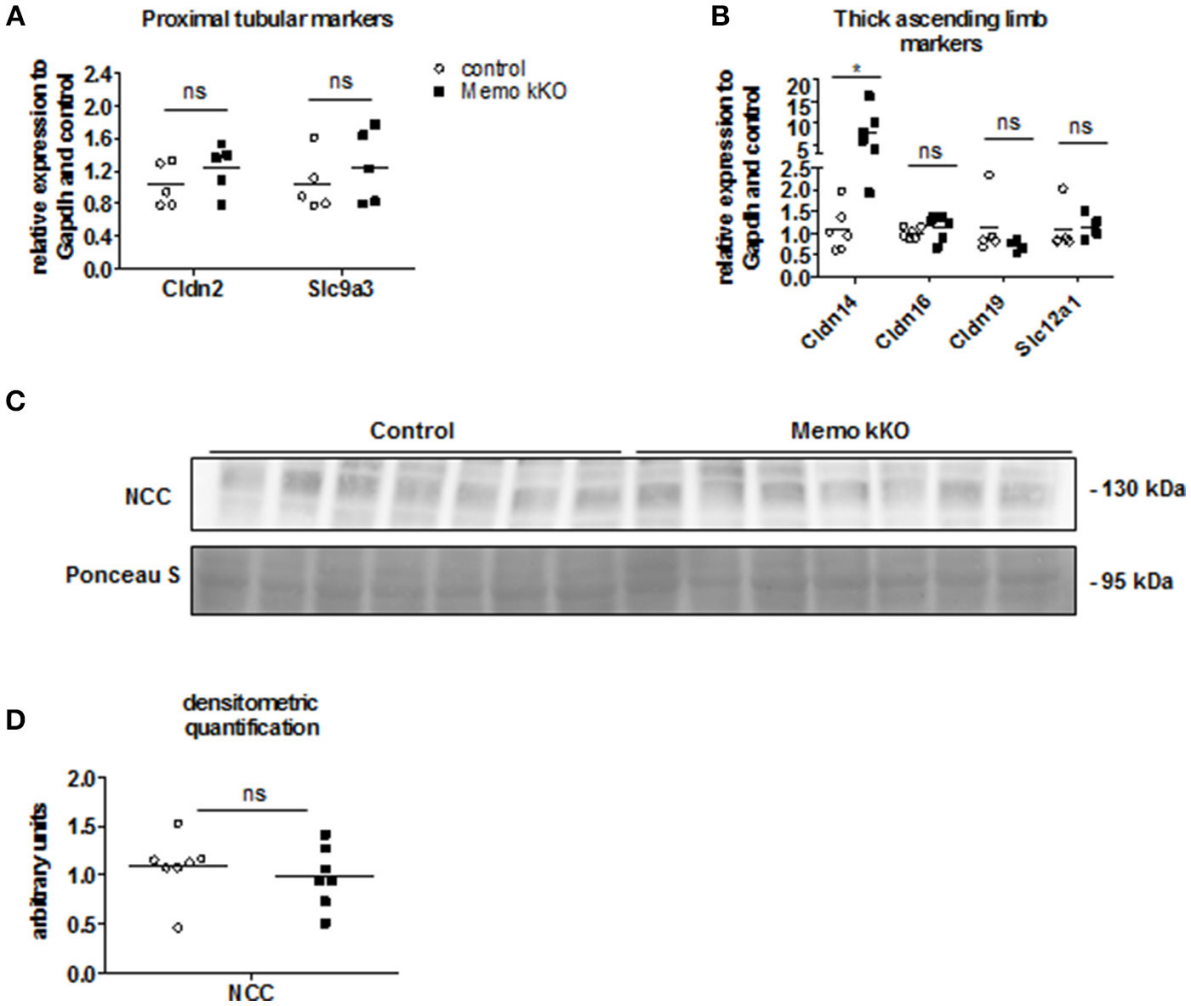
No evidence of DCT-CNT plasticity, but discrete alteration in proximal segments in Memo kKO. Transcripts of renal genes coding for proximal tubular proteins Claudin2 (*Cldn2*) and NHE3 (*Slc9a3*) were quantified by qPCR **(A)**. qPCR analysis of genes expressed in thick ascending limb of Henle are shown **(B)**. Western blot analysis of membrane-enriched protein preparations revealed comparable quantity of NCC in both genotypes **(C)**, quantification in **(D)**. Ponceau S in C is the same loading control as used in Figure 5B. NCC, sodium-chloride cotransporter. ^*^p < 0.05 (*t*-test); ns, not significant (*t*-test). *n* = 5 per genotype **(A)**; *n* = 6 per condition for Cldn14, Cldn16; 5 controls and 4 Memo kKO for Cldn19 and Slc12a1 **(B)**; *n* = 7 per genotype **(C,D)**.

As the expression levels of the distal renal tubular calcium transporters were increased in kidneys from Memo kKO animals, we speculated that this could be a compensation for more proximal tubular dysfunction. Gene expression of Slc9a3 coding for sodium-hydrogen antiporter 3 (NHE3) as a marker of proximal tubular function was comparable between genotypes (Figure 4A). Similarly, expression of *Cldn2* involved in paracellular calcium transport in this segment was unaffected (Figure 4A). Next, we investigated markers of calcium reabsorption in the thick ascending limb of Henle: gene expression of *Cldn16* and *Cldn19* that facilitate paracellular calcium reabsorption were comparable between the genotypes (Figure 4B). Only gene expression of their regulator *Cldn14* was increased (Figure 4B). *Slc12a1* coding for NKCC2 that is indirectly related to calcium transport (Figure 4B).

Finally, we asked whether the DCT-CNT segment might be overrepresented in the nephron of Memo kKO, thereby increasing the transcellular calcium transporters. However, we found no difference in the expression level of the sodium chloride cotransporter (NCC, expressed in DCT1) (Figure 4C, quantification in Figure 4D).

### Minor changes in phosphate homeostasis or intestine and bone calcium handling in memo kKo

**Figure 5:**
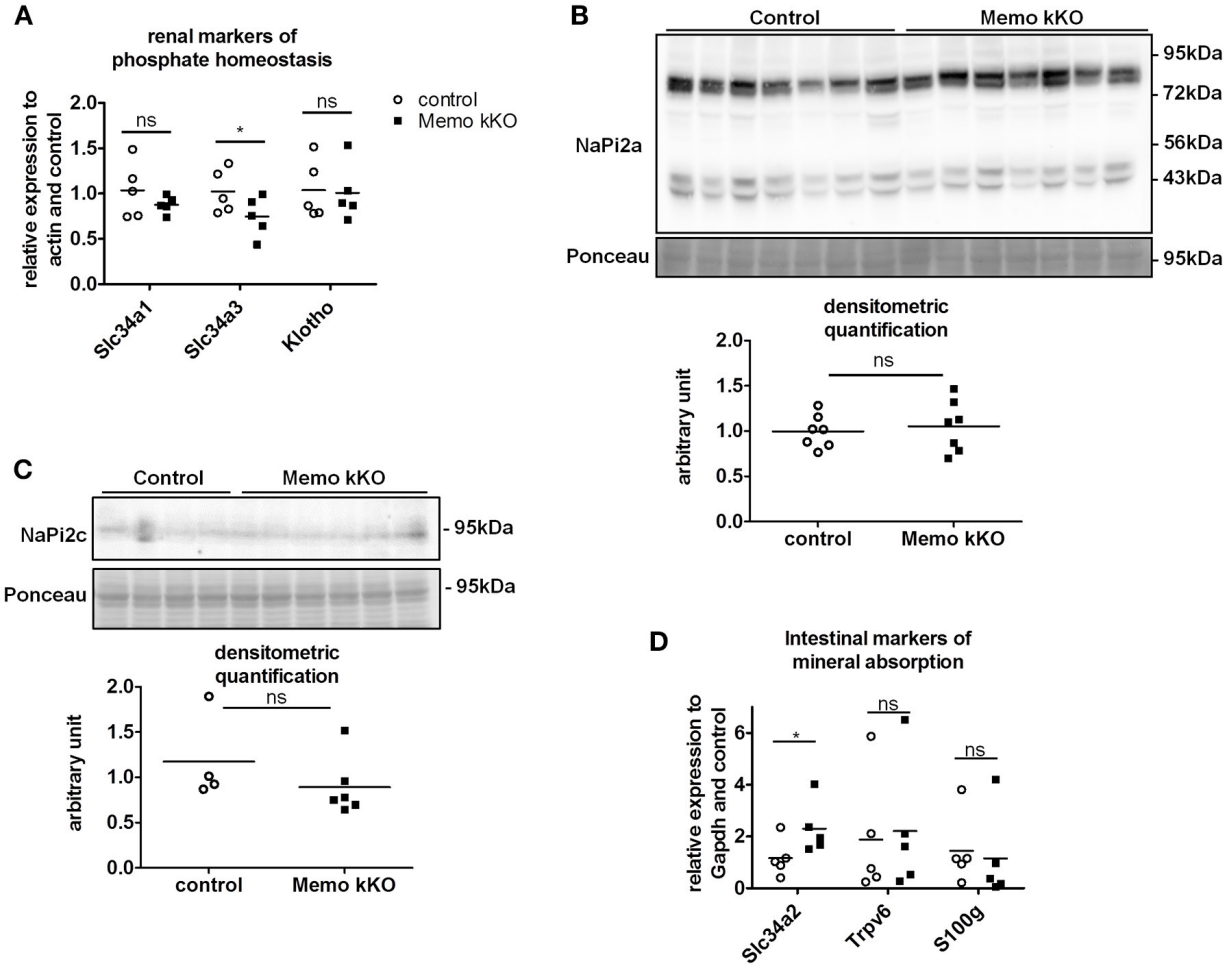
Minor changes in markers of renal and intestinal phosphate handling in Memo kKO. Transcripts of selected renal genes involved in phosphate homeostasis were assessed by qPCR **(A)**. Membrane protein abundance of renal NaPi2a was comparable between Memo kKO and controls **(B)**. NaPi2c was unchanged between genotypes in brush-border membrane enriched protein preparations **(C)**. *Slc34a2* expression in ileum was increased in Memo kKO compared to controls, whereas duodenal *Trpv6* and *S100g* transcripts were comparable between genotypes **(D)**. Ponceau S in B is the same loading control as used in Figure 4C. ^*^p < 0.05 (*t*-test). *n* = 5 per genotype **(A,B,D)**, *n* = 4 control and 6 Memo kKO **(C)**.

Due to the previously reported alterations in phosphate transporters in whole body KO Memo mice (Haenzi et al., 2014), we assessed phosphate homeostasis in the kidney-specific mouse model. Serum phosphate concentrations and urinary fractional excretion of phosphate were similar between Memo kKO and control mice (Table 2). We assessed transcriptional markers of renal phosphate handling and detected a decrease in *Slc34a3* coding for sodium-dependent phosphate cotransporter (NaPi)2c in Memo kKO (Figure 5A). *Slc34a1* transcripts coding for NaPi2a showed a trend to decreased expression in Memo kKO similar to what was observed in the whole body KO Memo mouse model (Haenzi et al., 2014), whereas *Klotho* was expressed at the same level in the two groups (Figure 5A). NaPi2a protein was assessed in membrane-enriched fractions and NaPi2c in brush border membrane-enriched preparations or kidney protein, and both showed no change between the two genotypes (Figures 5B,C).

Next, we screened for signs of intestinal alterations in mineral homeostasis as a compensation for the observed renal traits. We found that transcripts *Slc34a2* coding for NaPi2b were increased in ileum of Memo kKO compared to controls, whereas *Trpv6* and *S100g* (encoding Calbindin D9K) were comparable in duodenum of the two genotypes (Figure 5D).

**Figure 6:**
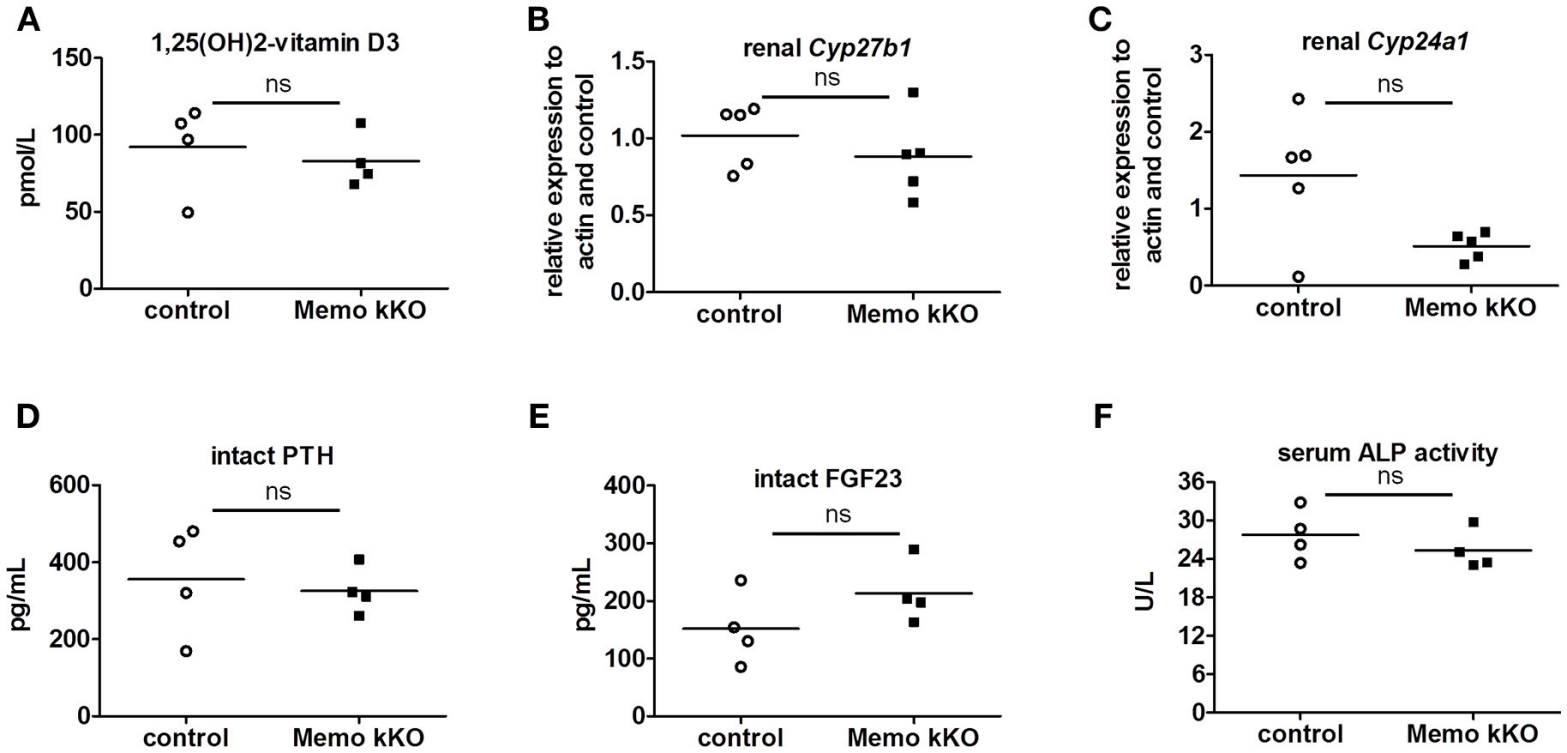
Normal regulators of mineral homeostasis in Memo kKO. Serum 1,25(OH)_2_**-**Vitamin D_3_ concentrations and gene expression of renal *Cyp27b1* and *Cyp24a1* were similar between genotypes **(A–C)**. We observed no change in serum intact PTH **(D)**, but a non-significant trend to increased FGF23 in Memo kKO **(E)**. Serum alkaline phosphatase (ALP) activity was comparable between genotypes **(F)**. ^*^p < 0.05 (*t*-test), *n* = 5 per genotype **(B–C)**, and sera of 4 animals pooled per data point, resulting in *n* = 5 × 4 animals per genotype **(A,D,E)**.

Systemic 1,25(OH)_2_-vitamin D_3_ concentrations and the mRNA expression levels of its two regulating enzymes *Cyp27b1* and *Cyp24a1* were comparable between genotypes (Figures 6A–C). Furthermore, intact parathyroid hormone (PTH) concentrations were similar (Figure 6D), and intact fibroblast growth factor (FGF)23 concentrations tended to increase in pooled sera from Memo kKO (Figure 6E). Serum alkaline phosphatase (ALP) activity, a marker of osteoblast activity that is dependent on both FGF23 and 1,25(OH)_2_-vitamin D_3_, was comparable between the genotypes (Figure 6F).

To complete the characterization of mineral homeostasis in these mice, we performed a micro-computed tomography analysis of L5 vertebrae. We found no difference in structural analysis of trabecular bone between Memo kKO and controls (Table 3).

**Table 3:** Micro-computed tomography analysis of L5 vertebrae from Memo kKO and controls.

	**Control**	**Memo kKO**	***t*-test**
	**Mean ± SD**	**Mean ± SD**	***p*-value**
n =	5	5	
age (d)	65.3 ± 2.3	66.7 ± 2.1	0.407
BMD (mg*cm^−3^)	202 ± 28	202 ± 19	0.976
BV/TV (%)	20 ± 3.5	20.3 ± 2.1	0.907
Tb.Th (mm)	0.059 ± 0.008	0.06 ± 0.001	0.895
Tb.N (mm^−1^)	3.4 ± 0.3	3.4 ± 0.3	0.942
Tb.S (mm)	0.18 ± 0.01	0.18 ± 0.01	0.524
Conn.D (mm^−1^)	146 ± 33	137 ± 28	0.630

*SD, standard deviation; BMD, bone mineral density; BV/TV, bone volume per total volume; Tb.Th, trabecular thickness; Tb.N, trabecular thickness; Tb.N, trabecular number; Tb.Sp, trabecular separation; Conn.D, connectivity density*.

Taken together, we detected discrete transcriptional changes in renal and intestinal phosphate transporters and a trend to increased serum FGF23 concentration in Memo kKO, which resembles, at a lower magnitude though, what has been reported for the whole body Memo KO mouse model (Haenzi et al., 2014).

### Effects of a vitamin D-deficient diet on memo kKo and control mice

As transcripts and protein abundance of renal calcium transporters present in the actively regulated DCT-CNT were increased in Memo kKO, we aimed at deciphering to which extent these abnormalities were dependent on vitamin D. For this purpose, we used two different experimental diets: a vitamin D-deficient diet (VDD) containing 0 IU/kg and a control diet containing 500 IU/kg vitamin D. Of note, the latter (control diet) contained less vitamin D (500 IU/kg) than the regular chow diet (1,600 IU/kg) used in the previous part of this study to compare kKO vs. control mice.

**Figure 7:**
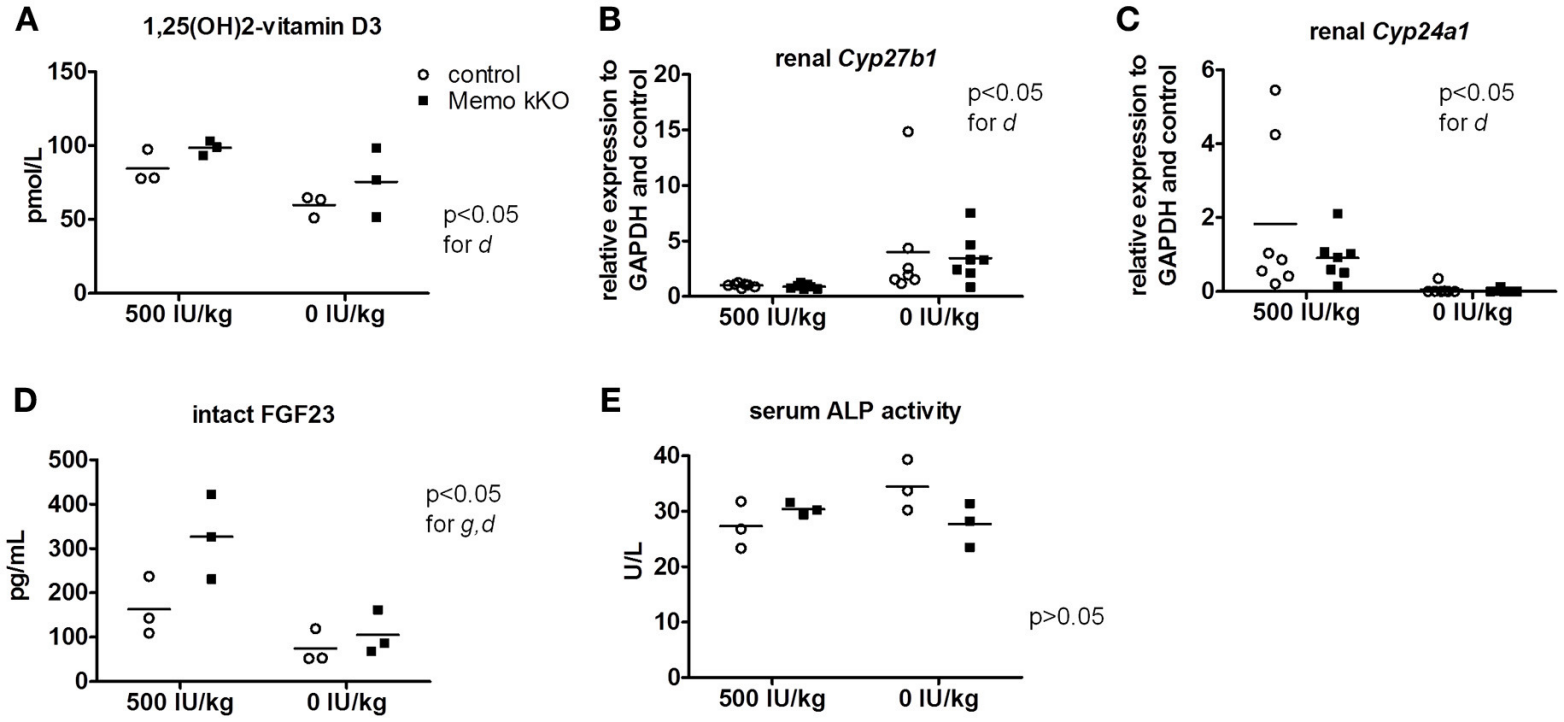
Effects of vitamin D deficient diet (VDD) on Memo kKO and control mice. Serum 1,25(OH)_2_**-**Vitamin D_3_ concentrations were affected by the diet **(A)**. Gene expression of renal *Cyp27b1* was increased **(B)** and *Cyp24a1* decreased **(C)** by VDD. Serum intact FGF23 concentrations were significantly different between genotypes and diets **(D)**. Serum alkaline phosphatase (ALP) activity showed tendency to increase in controls on vitamin D-deficient diet, not reaching significance though **(E)**. IU, international units. All results were analyzed by Two-way ANOVA. g, genotype effect; d, diet effect. Sera of 2 animals were pooled per data point, resulting in *n* = 3 × 2 animals per condition **(A,D,E)**. *n* = 7 animals per condition **(B,C)**.

After 5 weeks on experimental diets, control mice fed VDD showed a decline in serum 1,25(OH)_2_-vitamin D_3_ concentration (Figure 7A). An expected increase in renal transcription of *Cyp27b1* coding for 1α-hydroxylase was observed in mice of both genotypes fed VDD (Figure 7B). Conversely, expression of *Cyp24a1* coding for the 24α-hydroxylase was reduced under VDD (Figure 7C). However, measurements of electrolytes concentration in serum and in urines collected over 24 h in metabolic cages revealed only a discrete decline in serum creatinine by VDD, with no difference between the genotypes (Table 4). As another indirect sign of vitamin D deficiency, we observed lower serum concentrations of FGF23 under VDD (Figure 7D). Interestingly, the genotype was also significantly impacting on FGF23 concentration (Figure 7D). Serum ALP activity, which rises in case of vitamin D deficiency showed a non-significant trend to increased values in control mice on VDD compared to control mice, but not in Memo kKO (Figure 7E).

**Table 4:** Serum and 24 h urine analyses of control and Memo kKO mice exposed to 500 or 0 IU vitamin D per kg of food.

	**500 IU/kg**		**0 IU/kg**		**Two-way ANOVA**		
	**Vitamin D**		**Vitamin D**		***p*-value**		
	**Control**	**Memo kKO**	**Control**	**Memo kKO**	**Genotype**	**Diet**	**Interaction**
	**Mean ± SD**	**Mean ± SD**	**Mean ± SD**	**Mean ± SD**			
Body weight (g)	23.14 ± 2.14	22.14 ± 1.98	22.91 ± 1.69	23.02 ± 1.22	0.419	0.640	0.519
**SERUM ANALYSES**							
n =	3 × 2 pooled	3 × 2 pooled	3 × 2 pooled	3 × 2 pooled			
Creatinine (μM)	13.77 ± 1.97	13.37 ± 1.25	10.60 ± 0.85	10.77 ± 2.38^a^	0.806	0.035	0.919
Calcium (mM)	2.19 ± 0.05	2.21 ± 0.06	2.18 ± 0.09	2.14 ± 0.05	0.424	0.291	0.823
Phosphate (mM)	2.83 ± 0.57	2.56 ± 0.07	2.58 ± 0.38	2.39 ± 0.31	0.331	0.366	0.855
Sodium (mM)	155.3 ± 2.2	153.2 ± 1.1	153.3 ± 3.3	152.7 ± 3.9	0.662	0.470	0.423
Potassium (mM)	5.30 ± 1.21	4.77 ± 0.25	4.79 ± 0.15	4.82 ± 0.46	0.486	0.554	0.532
Albumin (g/L)	33.33 ± 1.15	34.00 ± 1	34.25 ± 0.66	34.33 ± 2.08	0.639	0.440	0.715
**ANALYSIS OF 24 H URINE COLLECTION**							
n =	7	7	7	7	
24 h urine volume (mg/d/gBW)	77.4 ± 31.1	66.7 ± 27.2	62.7 ± 32.9	69.8 ± 32.3	0.454	0.626	0.879
Osmolality (mOsm/kg)^b^	2620 ± 777	2634 ± 115	2855 ± 399	3482 ± 1380	0.517	0.285	0.534
Creatinine (μmol/d/gBW)	0.23 ± 0.03	0.21 ± 0.03	0.20 ± 0.03	0.20 ± 0.04	0.621	0.104	0.669
Calcium (μmol/d/gBW)	0.12 ± 0.03	0.15 ± 0.05	0.12 ± 0.05	0.10 ± 0.05	0.808	0.165	0.137
Phosphate (μmol/d/gBW)	8.48 ± 0.73	8.52 ± 1.16	8.04 ± 1.72	8.19 ± 2.00	0.874	0.503	0.919
Sodium (μmol/d/gBW)	14.25 ± 1.90	13.40 ± 2.24	12.31 ± 2.61	12.49 ± 2.78	0.720	0.131	0.578
Potassium (μmol/d/gBW)	21.29 ± 1.72	21.42 ± 4.07	19.28 ± 3.68	19.70 ± 4.84	0.848	0.201	0.919

SD, standard deviation; kKO, kidney-specific Memo KO.

a 1 value missing.

b n = 3 × 2 pooled per condition.

*Doxycycline treatment for 14 days starting at age 25–30 days; euthanasia 2–2.5 weeks after end of doxycycline treatment. IU, international unit. Values were normalized by body weight (BW) as indicated*.

To summarize, post-weaning depletion of vitamin D led to a state of mild 1,25(OH)_2_-vitamin D_3_ deficiency in both control and Memo kKO mice.

### Molecular changes in the distal tubule calcium transport system under vitamin D-deficient diet

**Figure 8:**
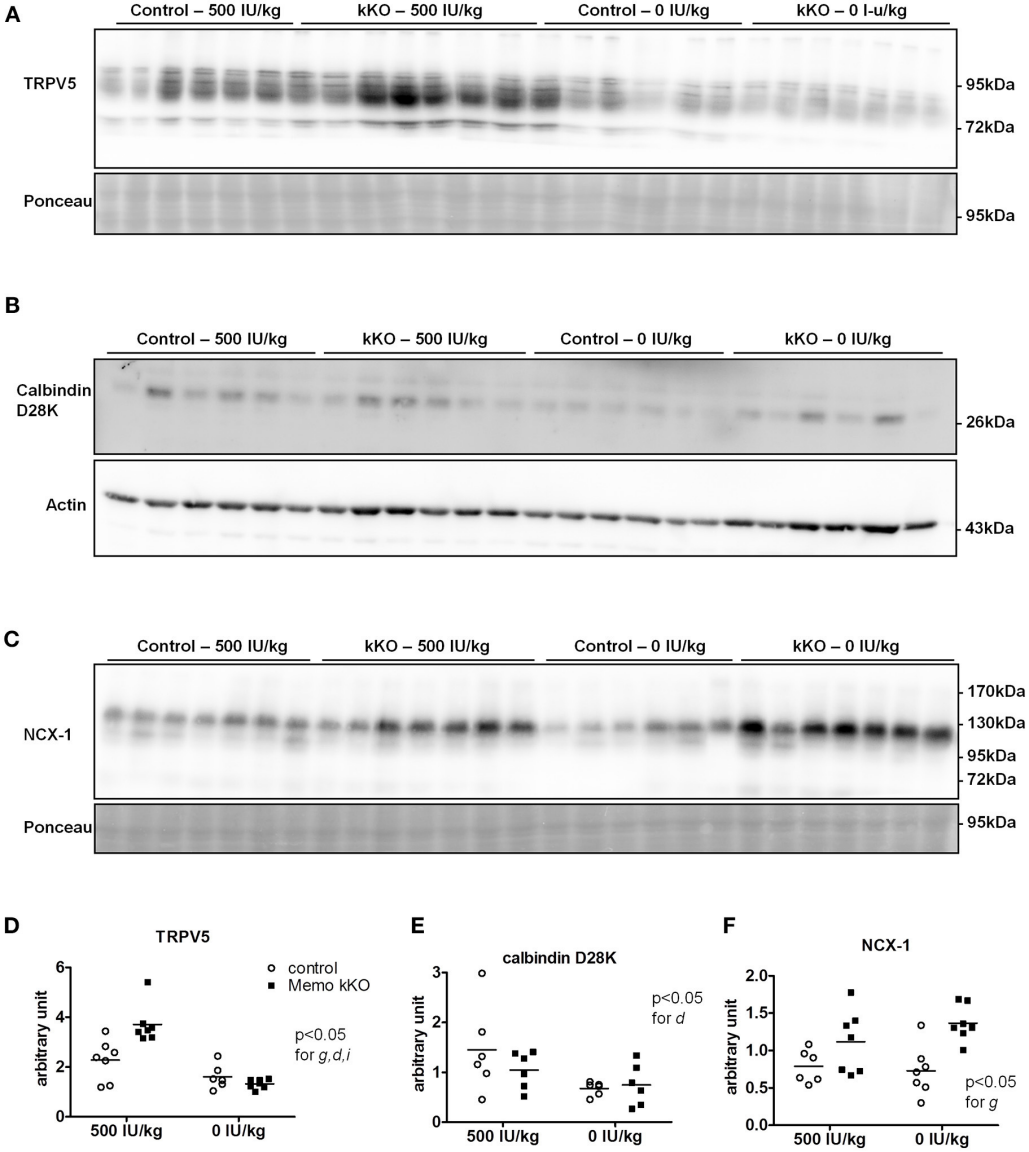
Renal TRPV5, but not NCX1 depend on dietary vitamin D in Memo kKO mice. Kidneys of mice fed 500 IU/kg vitamin D_3_ had higher membrane TRPV5 protein abundance by Western blot in Memo kKO, but this was abolished by a vitamin D-free diet **(A)**. Membrane NCX1 protein abundance remained elevated in Memo kKO kidney even after dietary vitamin D depletion; vitamin D deficiency caused a decrease in NCX1 membrane protein in control mice only **(B)**. No differences in renal calbindin D28K protein were observed **(C)**. **(D–F)**, Densitometric quantification and Two-way ANOVA analysis of **(A–C)**. g, genotype effect; d, diet effect; i, interaction between g and d.

We investigated protein expression levels of calcium transporters in mice fed the two experimental diets (0 and 500 IU/Kg) for 5 weeks. We found increased renal TRPV5 membrane protein abundance in kKO mice compared to control animals when both were on experimental control diet containing 500 IU vitamin D/kg (Figure 8A, quantification in Figure 8D). However, in mice fed VDD containing 0 IU/kg vitamin D, this increase was abolished (Figure 8A), indicating a role of vitamin D mediating the effect of Memo.

Under both VDD and control diet, no difference in renal calbindin D28K protein was observed between Memo kKO and controls (Figure 8B, quantification in Figure 8E).

For NCX1 membrane abundance, by contrast, the difference between genotypes seen in control diet persisted and even increased under VDD (Figure 8C, quantification in Figure 8F). In control mice, VDD slightly decreased NCX1 compared to control diets (Figure 8C).

**Figure 9:**
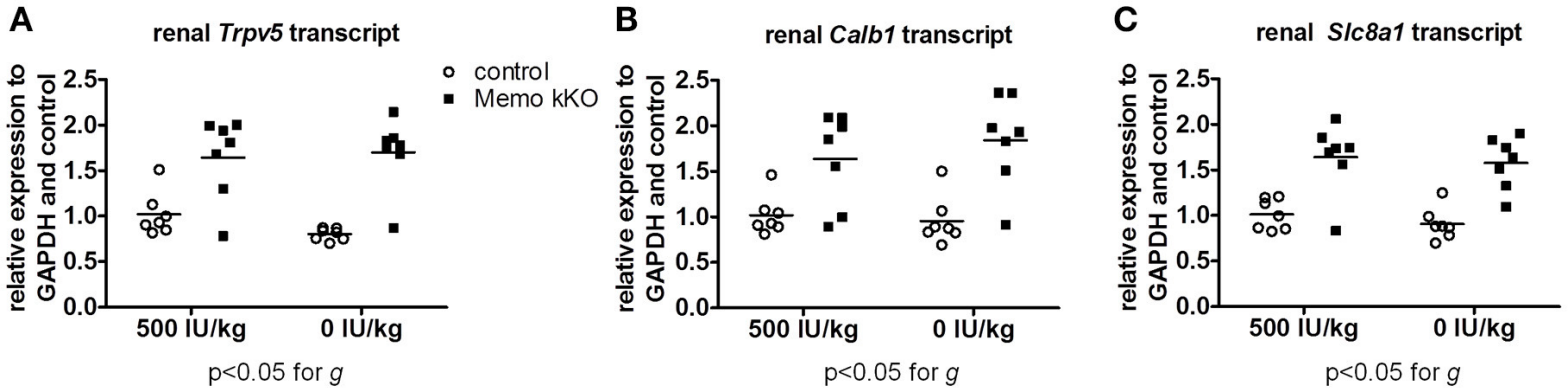
Gene expression of renal calcium transporters remained elevated in Memo kKO despite dietary vitamin D depletion. Gene expression of renal Trpv5 **(A)**, Calb1 **(B)**, and Slc8a1 **(C)** remained elevated in Memo kKO without significant diet effect as assessed by Two-way ANOVA. g, genotype effect.

Finally, we studied mRNA expression levels for the same three genes involved in calcium transport in the same animals. Whereas all renal *Trpv5, Calb1*, and *Slc8a1* transcripts were increased in Memo kKO compared to control mice in this set of animals, VDD had no detectable effect on any of the transporters' coding gene expression (Figure 9).

Collectively, we showed two distinct effects of dietary vitamin D content on the altered expression of calcium transporters in kKO Memo mice. On the one hand, the increased abundance of renal NCX1 membrane proteins was unaffected when Memo kKO mice were fed a vitamin D deficient diet. On the other hand, the increased expressions of renal TRPV5 and Calbindin D28K protein in Memo kKO were sensitive to vitamin D depletion and return to expression level close to the level measured in control mice. Transcription of the genes of these transporters were however not sensitive to VDD, pointing at a role of Memo in regulating post-transcriptional protein processing in the vitamin D regulating pathway.

## Discussion

We aimed at delineating the physiological involvement of Memo specifically in the adult kidney, without the influence of systemic factors encountered in whole body deletion of Memo. To this purpose, we have established a doxycycline-inducible kidney-specific Memo kKO mouse model that specifically abolishes Memo in renal tubular cells upon treatment by low-dose doxycycline.

The first finding to highlight is that these mice display normal morphology and have normal life expectancy. This is in sharp contrast with mouse models in which Memo is deleted in the whole body, even during adulthood and which develop premature aging and death. This indicates that deletion of Memo1 in tubular cells does not provide systemic signals responsible for the shorter life-span and premature aging observed in whole body Memo KO mice. Additionally, Memo kKO mice do not develop renal insufficiency, indicating that the rapidly progressive renal insufficiency observed in whole body Memo null mice is most probably due to circulating factors issued from extra-renal tissues or at least to the consequences of the deletion of Memo in other organs than the kidney or to non-tubular parts of the kidney.

Of note, the organ specificity of the kKO mouse model was robust as no leakage in the other organs tested was observed. More precisely, the previously reported low endogenous hepatic Pax8 promoter activity (Traykova-Brauch et al., 2008) has not affected total liver Memo protein in the current model and experimental protocol.

### Memo kKo mice vs. memo whole body KO

Memo kKO mice have slightly different and more moderate alterations of mineral metabolism compared to whole body Memo KO mice. In particular, Memo kKO mice did not show elevated calcemia or osteopenia in contrast to whole body Memo null mice (Haenzi et al., 2014; Moor et al., 2018). This is suggestive of a possible role of bone and/or intestinal Memo in modulating calcium mobilization. In the kidney, unbound calcium is freely filtered and subsequently reabsorbed along the renal tubules. Paracellular calcium transport in proximal tubule and thick ascending limb accounts for the majority of reabsorbed calcium, but hormone-regulated fine-tuning of calcium excretion takes place in the DCT-CNT (Moor and Bonny, 2016). There, vitamin D, PTH, sexual hormones, klotho and other factors regulate apical calcium entry through TRPV5, intracellular transport by calbindin D28 and calcium exit to the basolateral compartment through the sodium-calcium exchanger and the Calcium ATP-ase. We observed a significant increase in the transcription and in protein abundance of various DCT calcium transporters in Memo kKO mouse kidney, similar to what was previously reported for the whole body Memo KO mouse model (Haenzi et al., 2014). This highlights a direct role of renal tubular Memo in regulating renal calcium transporters in the DCT-CNT. In addition, renal claudin 14 gene expression was increased in Memo kKO without affecting claudin 16 and 19 transcripts. Further investigations will be required to uncover the cause and consequence of this finding.

Memo kKO mice and whole body Memo null mouse models showed varying trends of increased FGF23 serum concentrations (Haenzi et al., 2014; Moor et al., 2018). Additionally, Memo kKO mice fed the experimental 500 IU/kg vitamin D diet displayed significantly increased serum FGF23 levels compared to controls. This increase was however absent in Memo kKO mice on VDD, indicating that the increase in serum FGF23 levels in Memo kKO is dependent on 1,25(OH)_2_-vitamin D_3_ and suggesting a specific role of 1,25(OH)_2_-vitamin D_3_ in the Memo null phenotype.

Noteworthy, the present findings indicate that, although whole body Memo null mice partially reproduced premature aging features and insulin hypersensitivity phenotypes of Klotho-deficient mice (Haenzi et al., 2014), unlike the case of klotho (Lindberg et al., 2014), the kidney is not solely responsible for most of Memo physiological effects.

### Renal memo as a transcriptional repressor for genes involved in calcium transport

In absence of Memo, we observed increased mRNA expression of genes involved in transepithelial distal calcium transport. This was already reported for the whole body Memo KO mouse model (Haenzi et al., 2014) but was explained at that time by the increased concentration of 1,25(OH)_2_ vitamin D measured in this specific model. In the present kidney-specific model of Memo inactivation, and in absence of high levels of 1,25(OH)2 vitamin D or in changes of PTH, calcemia or phosphatemia, we showed that the transcription of *Trpv5, Calb1* and *Slc8a1*, but not *Atp2b4* is increased. Increased expression of these genes upon disappearance of renal Memo may arise from different causes: (1) The absence of Memo may alter DCT-CNT plasticity and may cause elongation or hypertrophy of the DCT-CNT segments resulting in a relative higher expression of these transporters in the kidney. (2) Renal Memo could affect 1,25(OH)_2_-vitamin D_3_ metabolism in the tubules and lead to higher local, but not systemic, vitamin D concentrations that would increase vitamin D-sensitive calcium transporters expression. (3) Because the final resulting calciuria is not affected: Loss of the redox protein Memo might disturb the function of the high energy dependent proximal tubular segment and lead to loss of proximal tubular calcium reabsorption that might induce downstream compensation by tubular crosstalk and increased calcium transport proteins in the DCT-CNT. (4) Finally, Memo could repress directly the transcription of certain genes.

Here, we tested several of these hypotheses. First, we verified that sodium-chloride cotransporter, which is expressed in the DCT1 and is unrelated to direct calcium reabsorption, had unchanged protein abundance. This does not support a putative tubular plasticity and elongation of the DCT-CNT segment induced by the absence of Memo. Second, we investigated a possible increase of 1,25(OH)2-vitamin D_3_, but did not find any difference, neither in circulating serum levels nor in the renal expression levels of *Cyp27b1* and *Cyp24a1* coding for the two renal enzymes involved in 1,25(OH)2-vitamin D synthesis and degradation, respectively. Third, we have found no evidence for a general dysfunction of the proximal tubule or thick ascending limb of Henle that would lead to downstream compensation by increased calcium absorption. And finally, a direct effect of Memo acting as repressor on certain gene expression remains a possibility to explore further in dedicated studies.

Importantly, vitamin D had no influence on the Memo-dependent increase of the transcription of these genes, as VDD was not associated with any decrease of the level of expression, even at 0 IU/kg vitamin D in the diet for 5 weeks. Whether the effect of Memo on the transcription of these genes is mediated by other partners or whether it is direct was not tested in the *in vivo* model and still remains unknown.

### Post-translational effects of vitamin D on memo-dependent protein expression

When we measured protein expression of the genes involved in DCT-CNT calcium reabsorption, we noted that protein expression level was increased in the same proportion as mRNA. We further explored the potential underlying mechanisms by modifying systemic 1,25(OH)_2_-vitamin D_3_ concentrations to a state of mild 1,25(OH)_2_-vitamin D_3_ deficiency. We exposed mice to experimental diets containing 0 or 500 IU/kg vitamin D for 5 weeks and first verified the expected physiological effects. We observed mirrored gene expression of the renal hydroxylases *Cyp24a1* and *Cyp27b1* and decreased FGF23 serum concentration. We also observed lower creatinine concentrations under vitamin D deficient diet (VDD), as has been reported before in humans (Fonseca et al., 1984).

To our surprise, the increase in renal TRPV5 and calbindin-D28K protein abundance in Memo kKO was abolished by VDD, suggesting that vitamin D may act at the post-translational level. By contrast, NCX1 membrane protein remained increased, even after 5 weeks of VDD. This indicates that some but not all calcium transporters protein expressions are regulated via 1,25(OH)_2_-vitamin D_3_ in Memo kKO mice and points to differential post-translational regulation of TRPV5 and NCX1 by vitamin D in the DCT-CNT. Accordingly, high calcium diets have previously showed similar discrepant regulation of NCX1 and TRPV5. Mice fed high calcium diet displayed lower TRPV5 and calbindin D28K expression, while NCX1 was left unaffected in wildtype mice, opposed to high calcium diet-induced upregulation of all three proteins in 25α-hydroxylase deficient mice (Hoenderop et al., 2002).

As the increase in renal NCX1 membrane abundance in Memo kKO was not decreased by VDD, an alternative explanation for NCX1 increase may be a challenged redox state in Memo kKO. Indeed, Shelton et al. showed increased *Slc8a1* transcripts coding for NCX1 in a renal transcriptome of mice deficient for *Nrf2* (Shelton et al., 2015)*. Nrf2* itself is a transcriptional regulator of oxidative stress responses, the ortholog of which being stimulated in *memo-1* deficient nematodes (Ewald et al., 2017).

At mild vitamin D deficiency (500 IU/kg, 5 weeks), the increased expression levels of genes coding for TRPV5, calbindin D28K and NCX1 remained unaffected. This indicates that vitamin D deficiency may first impact post-translationally on calcium transporters before affecting transporter transcription at higher degrees of vitamin D deficiency that would be severe enough to affect serum calcium levels (Hoenderop et al., 2001).

Interestingly, vitamin D has been previously shown to regulate proteins at the post-transcriptional level (An et al., 2010), in a ligand-dependent manner. FoxO proteins are deacetylated and dephosphorylated by 1,25(OH)2 vitamin D bound to its receptor, leading to more active FoxO genes. Similar mechanisms could be thought of for vitamin D regulating the post-transcriptional modulation of TRPV5, Calbindin-D28k and NCX1 and would explain the discrepancy observed between TRPV5 and NCX1. Lower TRPV5 protein expression in the DCT-CNT would theoretically lead to increased calciuria, but this was not observed and may have been compensated by lower calcium absorption in the intestine, or by increased calcium reabsorption in the proximal tubule or in the thick ascending limb of Henle.

This study contains some limitations. We subjected the current mouse model to a dietary intervention causing only a mild vitamin D_3_ deficiency without apparent hypocalcemia. However, in a presumed steady state after 5 weeks of treatment, changes of the relevant transport proteins of interest were already detectable. A further limitation is the use of doxycycline to induce the recombination, which may affect mitochondrial function (Chatzispyrou et al., 2015). Theoretically, doxycycline pharmacokinetics and renal toxicity could be different between genotypes when liver function is affected due to off-target inactivation of the gene of interest in the liver. However, we chose the lowest-possible doxycycline regimen to minimize this bias. Another limitation is the fact that the vitamin D deficient diet (0 IU Vitamin D/kg of food) was run in parallel with a low dose vitamin D regimen (500 IU vitamin D/kg food) and not directly with the normal chow (containing 1,600 IU vitamin D/kg food). This renders the interpretation of the data more complex and does not allow direct comparison between the 3 diets (1,600, 500, and 0 IU/kg food). Another limitation relates to the inactivation of Memo only in the tubular cells of the kidney and not to interstitial, glomerular or circulating cells. And finally, we did study only male mice and putative gender effect cannot be anticipated from the present data.

To conclude, we have unraveled an organ-specific function of Memo in the kidney that affects the expression of renal calcium transporters, mediated in part by vitamin D presence, and claudin 14. We also show here that TRPV5 and NCX1 protein expression are differentially regulated by vitamin D at the post-transcriptional level in Memo kKO mice, a finding that will need further exploration. These observations in Memo-deficient mice provide insights in differential mechanisms simultaneously regulating renal calcium transport molecules, which might prove useful for therapeutic efforts to circumvent mineral and electrolyte disorders.

## Author contributions

MM, BH, and OB: Participation in experimental design. MM, FL: Performed experiments. MM, FL, OB: Participation in data analysis. MM, BH, NH, OB: Participation in data interpretation. RK, NH: Provided laboratory materials. MM, OB: Wrote the manuscript. MM, BH, FL, RK, NH and OB: Critically read and commented on manuscript and agreed to manuscript submission.

### Conflict of interest statement

The authors declare that the research was conducted in the absence of any commercial or financial relationships that could be construed as a potential conflict of interest.
