# Supplementary figures and images for: Renal Memo1 Differentially Regulates the Expression of Vitamin D-Dependent Distal Renal Tubular Calcium Transporters
Source: Front Physiol. 2018 Jul 9;9:874. doi: 10.3389/fphys.2018.00874 (PMC6046545; doi:10.3389/fphys.2018.00874)

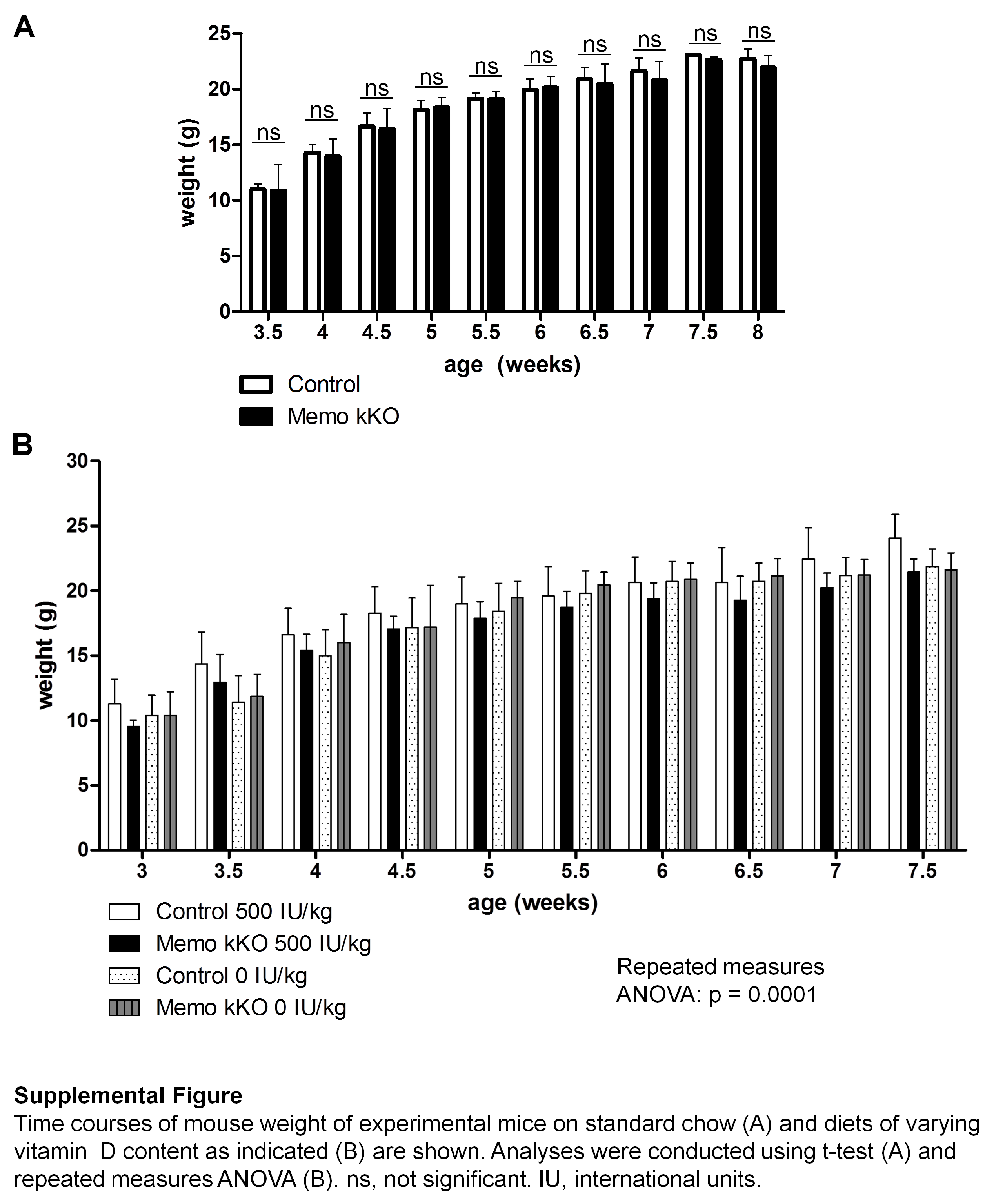

Supplement: Supplementary file 1 [file Image_1.tif]
